# Oxidative Phosphorylation: A Target for Novel Therapeutic Strategies Against Ovarian Cancer

**DOI:** 10.3390/cancers10090337

**Published:** 2018-09-18

**Authors:** Amruta P. Nayak, Arvinder Kapur, Lisa Barroilhet, Manish S. Patankar

**Affiliations:** 1 Indian Institute of Science Education and Research, Pune 411008, India; amruta.pn@students.iiserpune.ac.in; 2 Department of Obstetrics and Gynecology, University of Wisconsin-Madison, Madison, WI 54911, USA; akaur@wisc.edu (A.K.); barroilhet@wisc.edu (L.B.)

**Keywords:** high grade serous ovarian cancer, metabolism, mitochondria, oxidative phosphorylation, oxidative stress, biguanides, atovaquone, plumbagin, thiazolidinediones, ubiquinone, Nrf-2

## Abstract

Aerobic glycolysis is an important metabolic adaptation of cancer cells. There is growing evidence that oxidative phosphorylation is also an active metabolic pathway in many tumors, including in high grade serous ovarian cancer. Metastasized ovarian tumors use fatty acids for their energy needs. There is also evidence of ovarian cancer stem cells privileging oxidative phosphorylation (OXPHOS) for their metabolic needs. Metformin and thiazolidinediones such as rosiglitazone restrict tumor growth by inhibiting specific steps in the mitochondrial electron transport chain. These observations suggest that strategies to interfere with oxidative phosphorylation should be considered for the treatment of ovarian tumors. Here, we review the literature that supports this hypothesis and describe potential agents and critical control points in the oxidative phosphorylation pathway that can be targeted using small molecule agents. In this review, we also discuss potential barriers that can reduce the efficacy of the inhibitors of oxidative phosphorylation.

## 1. Introduction

Metabolic adaptations allow tumors to maintain a highly proliferative state. Evidence in support of such adaptations was obtained nearly a century ago by Otto Warburg, and Carl and Gert Cori and their colleagues when they demonstrated an increased uptake of glucose by tumors as compared to normal tissues [1,2]. Warburg further demonstrated that even when sufficient oxygen was available, tumors used glycolysis to metabolize glucose to lactic acid [3,4]. In this respect, glucose metabolism in tumor cells resembles that occurring under anaerobic conditions. However, because glucose was being metabolized to lactate in the presence of oxygen, Warburg coined the term “aerobic glycolysis” to accurately describe this metabolic process in tumors [3]. Although glucose breakdown through oxidative phosphorylation (OXPHOS) yields maximum number of ATP, curtailing the metabolism to glycolysis provides the necessary biomolecule precursors needed by the tumors to maintain a high level of proliferation [5,6,7,8]. Several key enzymes in the glycolytic pathway and tricarboxylic acid cycle, (pyruvate kinase M2, pyruvate dehydrogenase kinase, isocitrate dehydrogenase, succinate dehydrogenase, lactate dehydrogenase and others (representative articles include [9,10,11,12,13,14,15,16,17]) are targets for anti-cancer drugs. 

A rapidly growing body of evidence is demonstrating that an adaptation to aerobic glycolysis does not entail a complete shutdown of oxidative phosphorylation (OXPHOS) in tumors. Active electron transport occurs in cancer cells that trigger tumor recurrence and in cancer stem cells [18,19,20]. Here, we review evidence supporting the importance of OXPHOS in high grade serous ovarian cancer (HGSOC), discuss small molecule inhibitors of OXPHOS, their mechanism of action, and potential barriers to the use of such agents for the treatment of HGSOC. 

## 2. Oxphos As Target for Hgsoc Therapy

Ovarian cancer is classified into type I and II diseases [21,22,23]. Clear cell cancer and low grade endometrioid are major types of ovarian tumors classified as Type I malignancies with mutations in ARID1A (AT-rich interactive domain-containing protein 1A), K-Ras (Kirsten rat sarcoma) and PTEN (phosphatase and tensin homolog). High grade serous ovarian cancer (HGSOC), the predominant subtype, is classified as Type II disease and is characterized by mutations in p53 and copy number variations [24,25,26,27,28,29]. In the majority of the patients, HGSOC is detected at an advanced stage when the tumor has progressed to sites beyond the ovaries. While cytoreductive surgery and chemotherapy with platinum and taxanes are initially effective, they fail to prevent recurrence of HGSOC. Recurrent HGSOC responds poorly to most established and experimental therapies. While PARP (ADP ribose polymerase) inhibitors have extended overall survival [30,31,32,33], there remains a need for additional novel therapeutic approaches to treat HGSOC. In this review, we make the case that OXPHOS be considered as a druggable pathway while developing novel therapies against HGSOC. 

**Figure 1:**
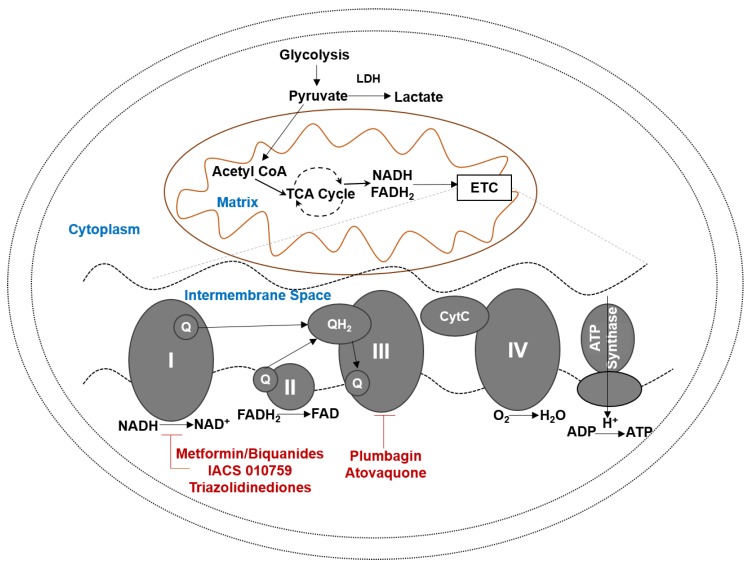
Oxidative phosphorylation. Aerobic glycolysis metabolizes glucose to lactic acid. Oxidative phosphorylation (OXPHOS) occurs in mitochondria and leads to efficient generation of ATP. OXPHOS is an active pathway in tumors and cancer stem cells. Several inhibitors or the various subunits of the mitochondrial electron transport complexes can serve as candidates for tumor therapy. Prominent drug candidates for HGSOC are shown. CytC, Cytochrome C, ETC, Electron Transport Chain, LDH, Lactate Dehydrogenase, Q, unbiquinone, QH2, Ubiquinol, TCA, Tricarboxylic Acid Cycle, FADH_2_, Flavine Adenine Dinucleotide, NADH, Nicotinamide Adenine Dinucleotide.

In normal cells, glucose is metabolized through glycolysis, tricarboxylic acid cycle and OXPHOS to produce 34–38 molecules of ATP per molecule of glucose (Figure 1). In cancer and other highly proliferative and activated cells (immune cells, for example), the end product of glycolysis, pyruvate, is not transferred to the mitochondria and consumed in the tricarboxylic acid cycle, but instead is converted to lactate (Figure 1). This conversion to lactate allows the cells to regenerate NAD (Nicotinamide Adenosine Dinucleotide) needed to drive the conversion of glyceraldehyde-3-phosphate to 1,3-bisphosphoglycerate in glycolysis.

An active glycolytic pathway supplies the biochemical precursors required for protein, nucleotide and lipid synthesis. This is an important reason why cancer cells limit glucose metabolism to glycolysis even when there is sufficient availability of oxygen. Ovarian tumors also show metabolic adaptation to aerobic glycolysis that allows them to maintain an increased proliferative capacity and survive under anchorage dependent conditions [34,35,36]. Adaptation of the ovarian cancer cells to aerobic glycolysis is supported by the increased expression of the glycolytic enzymes pyruvate kinase isoform M2 (PKM2), hexokinase II, and lactate dehydrogenase A (LDHA). PKM2 catalyzes the conversion of phosphoenolpyruvate to pyruvate and regulates the flux of acetyl coA available to enter the tricarboxylic aid cycle. Elevated expression of PKM2 correlates with decreased progression free survival in HGSOC although overall survival is not affected [37]. Hexokinase II is also upregulated in HGSOC [38,39]. Elevated hexokinase II expression contributes to chemoresistance in ovarian tumors [40]. 

The metabolic shift to aerobic glycolysis provides the precursors for synthesis of proteins, nucleotides and lipids, at the expense of ATP. To compensate, cancer cells overexpress glucose transporters. As a result, there is an increased uptake of glucose that is catabolized through aerobic glycolysis. Serous ovarian tumors express high levels of glucose transporters GLUT1, GLUT3 and GLUT4, as compared to healthy and benign ovarian tissues [41,42]. Increased uptake of glucose through the upregulation of glucose transporters (GLUT) is a hallmark of HGSOC allowing their imaging by 18-fluoro-deoxyglucose positron emission tomography (FDG-PET) [43,44]. 

The molecular mechanisms responsible for the metabolic reprogramming in cancer are under investigation. One mechanism is through the regulation of pyruvate kinase M2 isoform (PKM2) activity through its interactions with growth factor receptors [45]. There is emerging evidence that mutated *BRCA1* [46] and mutated p53 (reviewed in [47]), two genes that are most frequently mutated in HGSOC, also contribute to the shift to aerobic glycolysis. In HGSOC, the sulfatase, h-Sulf-1 is downregulated [48]. Loss of h-sulf-1 in HGSOC increases glycolytic activity through the phosphorylation of pyruvate dehydrogenase causing a decrease in availability of pyruvate in the tricarboxylic acid cycle [49]. All of these results clearly demonstrate that glycolysis is a major metabolic adaptation occurring in HGSOC. 

### 2.1. Relevance of OXPHOS in Solid Tumors 

While aerobic glycolysis is an important adaptation in HGSOC, OXPHOS is also an active pathway in cancer cells [50,51,52,53,54,55,56]. Initial data in support of this observation was gained from experiments with the tetracycline-inducible K-Ras (G12D) mouse model for pancreatic cancer [57]. Withdrawal of doxycycline caused regression of the pancreatic ductal carcinomas [55]. However, tumor recurrence was observed in the mice 4–5 months after doxycycline withdrawal. Tumor relapse was attributed to cancer stem cells surviving the ablation of mutant K-Ras. These surviving cancer stem cells had increased mitochondrial biogenesis with higher OXPHOS activity but impaired glycolysis. The relapsing tumors were responsive to the OXPHOS inhibitor, oligomycin [55]. 

Tumor cells that utilize aerobic glycolysis, coexist with cancer cells with active OXPHOS. A recent report by Yu et al [58] developed a model to predict the predominant metabolic pathway utilized by normal and cancer cells. Glycolysis is indicated by high expression of HIF-1α (Hypoxia inducible factor-1α) and low levels of phospho-5’ AMP-activated protein kinase (pAMPK), whereas OXPHOS-reliant tumors have low levels of HIF-1α and high levels of pAMPK. Some cancer cells express high levels of both HIF-1α and pAMPK indicating active glycolysis as well as OXPHOS. 

There are, however, some indicators that active mitochondrial metabolism may have a favorable outcome. The Bioenergetic Cellular (BEC) index, a ratio β-F1ATPase (F1 portion of adenosine triphosphate synthase) to HSP60 (Heat Shock Protein 60) and GAPDH (Glyceraldehyde 3-Phosphate dehydrogenase) expression, predicts the metabolic state of a cell [59]. A higher BEC is an indicator of active OXPHOS. In one study, thirty six of 55 HGSOC patients had a BEC of less than 2.65 [60]. Progression free survival was higher in patients with <2.65 compared to the 19 patients with higher BEC (9.8 versus 5.3 months). However, the BEC does not account for metabolic heterogeneity and therefore these observations do not rule out the presence of tumor foci with active OXPHOS. There is evidence that agents targeting OXPHOS can be used to target cancer initiating cells, chemoresistant tumors as well as non-tumor cells from the tumor microenvironment. 

### 2.2. Reliance of Ovarian Cancer Stem Cells on OXPHOS

Perhaps the largest impact of OXPHOS is in the survival and proliferation of cancer initiating stem cells. Tumor initiating cells isolated from tumorigenic murine ovarian surface epithelial (MOSE) cells showed increased expression of glucose transporters and an overactive glycolytic pathway [61,62]. However, these tumor initiating cells also had a higher capacity than non-tumor initiating tumor-forming MOSE cells for mitochondrial oxygen consumption. The tumor initiating MOSE cells also exhibited higher survivability when cultured in media that did not contain glucose but was supplemented with glutamine and fatty acids. The observation that the tumor initiating cells are better able to survive on glutamine-supplemented media suggests that they are less dependent on glycolysis and, through the entry of glutamine in the tricarboxylic acid cycle are able to generate sufficient NADH (Nicotinamide Adenine Dinucleotide) and FADH2 (Flavine Adenine Dinucleotide). The higher mitochondrial capacity facilitates production of sufficient levels of ATP that drive their proliferation.

CD44^+^/CD117^+^ cancer stem cells isolated from the peritoneal fluid of HGSOC patients that had the ability to form tumors in mice, showed decreased levels of pyruvate dehydrogenase kinase (PDHK1) and increased expression of isocitrate dehydrogenase (IDH2) [18]. This observation is in stark contrast to the non-cancer stem cells (CD44^+^/CD117^−^) from HGSOC patients where the PDHK1 was upregulated and IDH2 was significantly lower. PDHK1 negatively regulates pyruvate dehydrogenase and as a result controls the amount of acetyl-CoA (Coenzyme A) available for the tricarboxylic acid cycle. The decrease in PDHK1 and increase in IDH2 in the CD44^+^/CD117^+^ ovarian cancer stem cell population are indicators of enhanced tricarboxylic acid cycle. The CD44^+^/CD117^+^ cancer stem cells produced higher levels of oxygen radicals and had enhanced OXPHOS than the non-stem cell (CD44^+^/CD117^−^) population. RAG2^−/−^ mice implanted with HGSOC tumors when maintained on a diet supplemented with the glycolysis inhibitor, 2-deoxyglucose, instead of glucose, showed a decrease in tumor size. However, the surviving tumors from these mice were enriched in CD44^+^/CD117^+^ cancer stem cells [18].

### 2.3. OXPHOS in Chemoresistant HGSOC

There is also evidence that OXPHOS is an important pathway to target in chemoresistant tumors. Tumor necrosis factor receptor-associated protein 1 (TRAP1) is a mitochondrial chaperone from the Hsp90 family [63]. Increase in TRAP1 expression elevates aerobic glycolysis in ovarian cancer cell lines [64]. HGSOC lines with lower expression of TRAP1 or silencing of this gene increased the oxygen consumption rate and decreased extracellular acidification (a measure of aerobic glycolysis) [65]. Low expression of TRAP1 results in higher reliance on OXPHOS and is associated with resistance to platinum [64]. Chemoresistant ovarian cancer cells show increased OXPHOS activity and survive under limiting glucose levels or when the resistant tumors were implanted in mice that were fed 2-deoxyglucose [34]. 

### 2.4. OXPHOS and the Tumor Microenvironment

Tumor cells produce high levels of lactic acid in the tumor microenvironment. While the lactic acid can be transported by cancer cells through monocarboxylic acid transporters (MCTs) and used to promote tumor proliferation, the effect of the acidic environment on the non-malignant cells is also an important factor to consider when determining the metabolic profile of the tumor. For example, the lactic acid in the tumor microenvironment can regulate the activity of immune cells infiltrating the tumor microenvironment (reviewed in [66]). 

Such crosstalk in the tumor microenvironment is not unidirectional as the tumor cells can also be affected by the metabolic status of the fibroblasts from the microenvironment. For example, Ras (glycine at position 12 mutated to valine) mutations alter metabolism in cancer cells and increase the release of oxygen radicals [67]. These radicals induce oxidative stress in the intratumoral stroma, forcing a catabolic state that produces lactate, ketones, glutamine and fatty acids that serve as fuel to the cancer. 

Stromal cells from the tumor microenvironment express low levels of caveolin 1 and high MCT4 allowing them to expel lactate into the tumor microenvironment [68,69,70]. In a recent study, patients with *BRCA1* mutated breast cancers were treated with the anti-oxidant, *N*-acetyl cysteine [71]. Pathological examination showed a reversal in the expression of caveolin 1 and MCT4 by the stromal cells suggesting that neutralization of the oxygen radicals can inhibit the symbiotic relationship between the cancer cells and the stromal cells in the microenvironment. Additionally, this study also observed a decrease in ki67 stained cancer cells. Since the mitochondria are the major source for oxygen radicals, it can be argued that the stromal cells from the tumor microenvironment are OXPHOS-active and the oxygen radicals generated by these cells promote the proliferation of cancer cells. Therefore, inhibitors of OXPHOS can not only be successful because of their direct cytotoxic effects on cancer cells but also through the potential modulation of metabolism in stromal and other non-cancer cells from the tumor microenvironment. 

## 3. OXPHOS Provides Multiple Targets for Drug Development

High energy electrons from NADH and FADH_2_ are harvested in OXPHOS and transferred to molecular oxygen. Four multiprotein complexes located in the inner membrane of the mitochondria (Complexes I-IV) are required for electron transport (Figure 1). Electrons from NADH and FADH_2_ are extracted in complex I and complex II, respectively. Electrons from complex I and II are delivered to complex III via the electron carrier, ubiquinone (coenzyme 10, CoQ10). The quinone head group of CoQ10 participates in two electron redox reactions. Addition of one electron to CoQ10 yields semiquinone and further reduction of this intermediate leads to formation of ubiquinol. The electron transfer from NADH/FADH_2_ to ubiquinone occurs at the ubiquinone and ubiquinol binding sites, Q_0_ and Q_i,_ in the mitochondrial complexes I-III. Electron transport from complex III to complex IV is aided by cytochrome C (Cyt C). In complex IV, the electrons are delivered to molecular oxygen to form water. 

The transfer of electrons is coupled with pumping of protons from the matrix to the intermembrane of the mitochondria. This transfer of protons leads to the maintenance of a proton gradient (ΔΨ_pion_). The proton efflux from the matrix helps maintain a negative charge in the matrix and contributes to an electrical potential gradient (ΔΨ_m_). The electromotive force generated through proton transport provides the energy necessary for the fifth mitochondrial complex, the ATP synthase, to convert ADP to ATP. 

Complex chemical reactions and biochemical control points are required to regulate OXPHOS. From the standpoint of cancer drug discovery, this situation provides opportunities for the development of novel therapeutic strategies. Agents that interfere with electron transport, maintenance of the proton gradient (ΔΨ_pion_ and ΔΨ_m_) and transfer of electrons to oxygen and ATP synthesis can be developed as cancer therapeutics. While small molecules are likely the preferred agents to target OXPHOS, efforts are also underway to develop peptides that can specifically target this mitochondrial metabolic pathway (reviewed in Reference [72]). In the subsequent sections, we will discuss small molecule agents that interfere with OXPHOS. 

## 4. OXPHOS Inhibitors

### 4.1. Complex I Inhibiting Biguanides

Metformin and proguanil are biguanides with complex I inhibitory activities (Figure 1). Regular use of metformin reduces risk of ovarian cancer (OR 0.61, 95% CI 0.3–1.25) [73]. Nearly 70% of HGSOC patients using metformin survived for 5 years. In comparison, only 47% of HGSOC patients who were not on metformin survived for 5-years or more [74]. Romero and colleagues have analyzed the positive benefits of metformin use in HGSOC patients with diabetes. Approximately 51% of the diabetic patients who regularly used metformin had progression-free survival at 5-years post initial diagnosis of the cancer. In contrast, 23% of the nondiabetic metformin users and only 8% of non-diabetic non-metformin users had progression free survival at 5-years postdiagnosis [75]. The overall survival at 5-years post initial diagnosis of HGSOC was reported to be 63%, 37% and 23%, respectively, for these three cohorts [75]. 

Metformin inhibits complex I and thereby reduces ATP production. As a result of decreased ATP levels, AMPK is activated in cancer cells along with inhibition of mTORC1 (mammalian Target of Rapamycin Complex). Millimolar concentrations of metformin are required to inhibit complex I activity and there remains an active question of whether such high levels of metformin can be achieved in solid tumors. Proguanil inhibits complex I activity in the malarial parasite and is therefore administered in conjunction with atovaquone, a complex III inhibitor. However, proguanil has limited effect on human complex I and is therefore not suitable for cancer therapy [76,77]. Another bigiuanide, phenformin triggers lactic acidosis and therefore has major clinical toxicity. 

A novel agent, IACS-0107059, that likely mimics the biguanide functional group has been investigated as therapy for acute myeloid leukemia [78,79,80,81,82]. This compound blocks complex I at subnanomolar-nanomolar range and inhibits proliferation of HGSOC cells. Clinical trials are currently underway to test this compound against AML and solid tumors.

Eight clinical trials are currently posted in *clinicaltrials.gov* to test the effect of metformin in ovarian cancer patients. The majority of these trials are evaluating the combination of metformin with chemotherapy and are currently recruiting patients. Results from one trial (NCT01579812) showed that metformin was well tolerated. Ex vivo evaluation of the tumors showed significant decrease in viable cancer stem cell population. Additional studies are needed to determine if these positive benefits are due to the OXPHOS inhibitory effects of metformin. As demonstrated by Yu et al. [58], monitoring pAMPK and HIF-1α levels in the metformin clinical trials can potentially be used as biomarkers for the status of OXPHOS versus aerobic glycolysis in tumors providing insight into the metabolic adaptations occurring in the tumors in response to this biguanide. 

### 4.2. Oxidative Stress Inducers

**Figure 2:**
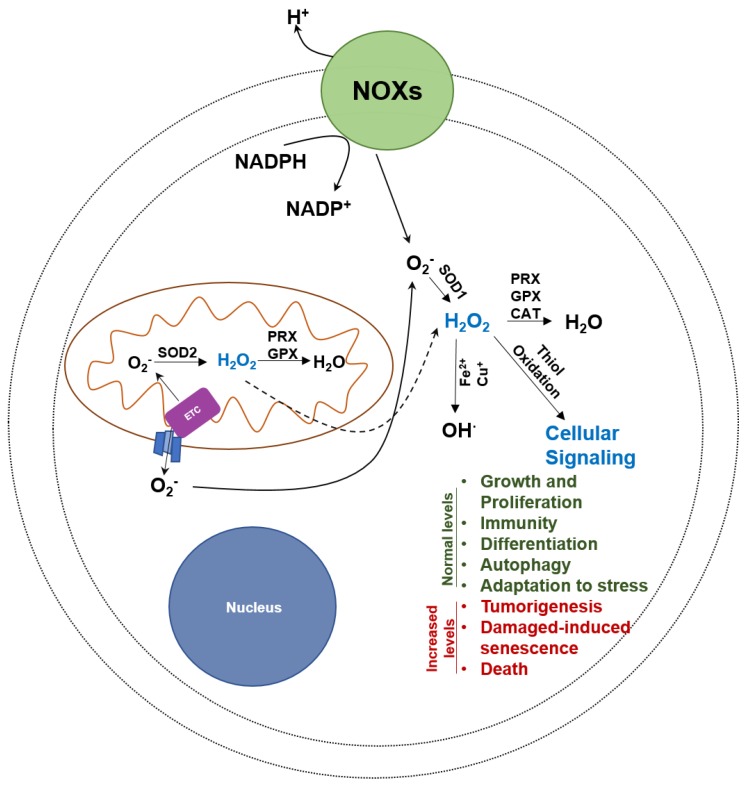
Uncontrolled oxidative stress is cytotoxic to cancer cells. OXPHOS is a major producer of oxygen radicals. While oxygen radicals have positive benefits in cells, a rapid and uncontrolled rise in hydroxyl radicals can lead to cancer cell death. PRX, peroxiredoxin, GPX, glutathione peroxidase, CAT, catalase, SOD, superoxide dismutase, and NOX, NADPH oxidase.

Oxidative stress results from an imbalance between the processes responsible for generation and sequestration of reactive oxygen radicals (ROS) [83]. Since the transfer of electrons to molecular oxygen is an integral step of the electron transport chain, the OXPHOS pathway is a major generator of oxygen radicals (Figure 2). A rapid increase in intracellular levels of oxygen radicals causes cellular damage and cell death. Atovaquone, a Food and Drug Administration (FDA) approved anti-malarial agent that inhibits complex III activity, is being repurposed for treatment of solid tumors [84,85,86].

Unpublished results from our group are demonstrating that atovaquone should be investigated for treatment of HGSOC. The naphthoquinone unit of atovaquone engages in redox reactions and interferes with electron transport mediated by ubiquinone [84,85,86]. 

There are several naturally occurring and synthetic molecules that contain the quinone, naphthoquinone or anthroquinone head groups. Plumbagin and juglone are examples of such compounds. Treatment of HGSOC cells with plumbagin results in an immediate increase in intracellular oxygen radical flux [87]. Plumbagin also inhibits oxygen consumption rate, decreases ATP synthesis and increases the redox ratio (NADH/FAD) and extracellular acidification rate (ECAR) [87]. These results indicate that plumbagin is likely to be an inhibitor of mitochondrial electron transport. 

Molecules that interfere with ubiquinone-mediated electron transport induce severe oxidative stress. There are at three major types of reactive oxygen species (superoxide anion O_2_^−^, hydrogen peroxide H_2_O_2_ and hydroxyl radicals OH^∙^) that are formed due to incomplete transfer of electrons to molecular oxygen (Figure 2). 

### 4.3. Superoxide Anion (O_2_^−^)

The primary source of superoxide anions is the electron transport chain in the mitochondria [88,89]. Leakage of electrons travelling through the multiple complexes in the electron transport chain results in one-electron reduction of oxygen to produce the superoxide anions. The second major producer of the superoxide anion are the NADPH oxidases (NOXs) which are transmembrane enzymes present at the different membranes in the cell (Figure 2) [90]. Superoxide anions are restricted in the cellular damage they can cause because they typically only react with peptide epitopes located near the iron sulfur complexes and hence do not cause indiscriminate cellular damage [91].

### 4.4. Hydrogen Peroxide (H_2_O_2_)

Reduction of the superoxide anions by superoxide dismutases (SODs) yields hydrogen peroxide (H_2_O_2_). H_2_O_2_, existing at nanomolar concentration in the cell, is the main ROS signaling molecule of the cell. It functions by oxidizing the thiolate anion (Cys-S^−^) of a cysteine to its sulfenic form (Cys-SO^−^). Oxidation of the cysteine thiol affects the formation of Inter- and Intramolecular disulfide bonds and has serious consequences on the biological properties of proteins [92]. This oxidation is reversed by the enzymatic action of thioredoxin (TRX) and glutaredoxin (GRX), which themselves are reduced back by thioredoxin reductase (TR). These set of enzymes essentially constitute the main group of molecules executing the redox signaling in the cell [93]. At abnormally high concentrations of H_2_O_2_ (as those observed during oxidative stress), the sulfenic form (Cys-SO^−^) is irreversibly oxidized to higher oxidized states of sulfinic (Cys-SO_2_^−^) and sulfonic (Cys-SO_3_^−^), which cannot be repaired through redox control and hence can cause significant cellular damage.

### 4.5. Hydroxyl Radical (OH^∙^)

The hydroxyl radical (OH^∙^) is the most reactive of the three ROS molecules described in this section. H_2_O_2_ reacts with metal cations (Fe^2+^, Cu^+^) present in the cytosol in a reaction called Fenton reaction, to produce OH^∙^. Additionally, nitric oxide synthases (NOS) also produce OH^∙^ along with NO_2_^∙^ under limiting concentration of cofactors and co-substrates. OH^∙^ reacts indiscriminately with various substrates such as lipids, proteins and DNA and leads to genomic instability [94]. Presence of OH^∙^ is abnormal and therefore, an indicator of high oxidative stress in the cell.

Oxidative stress induces pleiotropic effects in the cells. These include, but are not restricted to, activation of p53, inhibition of NFκB (Nuclear Factor kappa-B), activation of protein kinases and other signaling molecules, and decrease in the expression of survivin. Molecules such as plumbagin that increase intracellular oxygen radicals, are often thought to mediate pleiotropic effects that culminate in cancer cell death. However, it is important to consider that such molecules may also be specific in their ability to compete with ubiquinone and inhibit electron transport in the mitochondria and can therefore serve as important OXPHOS-targeting agents. 

## 5. Barriers to Using OXPHOS Inhibitors for HGSOC Therapy

### 5.1. Potential Toxicity of OXPHOS Inhibitors

Our studies with plumbagin clearly show that inhibition of electron transport results in a rapid increase in harmful oxygen radicals that cause significant cellular damage [87]. With OXPHOS serving as a major mechanism for energy generation, there is significant risk that inhibitors of this pathway may damage healthy tissues. Toxicity of the OXPHOS inhibitors is therefore a major concern that may curtail their use for the treatment of HGSOC and other tumors. It should be noted, however, that plumbagin did not produce major toxicity in pre-clinical studies in mouse models [95,96]. Additionally, metformin is generally a safe drug with minimum toxicity. While proguanil is effective inhibitor of the OXPHOS pathway in the malarial and other parasites, its specific activity as OXPHOS inhibitor is reduced in human cells [76,77]. This experience with atovaquone and proguanil suggests that rational drug development approaches can be applied to develop inhibitors that have higher potency against human OXPHOS complexes. Given the higher susceptibility of cancer cells to oxidative stress, well-designed and more potent inhibitors can potentially be used at lower concentrations to produce optimum activity in tumors while reducing toxicity in healthy tissues. Additionally, the OXPHOS inhibitors can also be functionalized with folate and other tumor targeting moieties to facilitate selective delivery of these agents to the tumor, thereby achieving higher efficacy with lower toxicity. 

### 5.2. Anti-Oxidant Mechanisms and Chemoresistance

There are elaborate antioxidant mechanisms to maintain steady state levels of oxygen radicals in all cells. Superoxide dismutase, catalase, peroxiredoxins, glutathione, glutathione reductase, thioredoxins and others form the network of anti-oxidant mechanisms that control oxidative stress. This network is controlled by a master regulatory transcription factor, Nrf-2. Cancer cells respond to inhibition of complex III by atovaquone, (unpublished observation) and plumbagin [87] by increasing the expression of Nrf-2. Therefore, the oxidative stress triggered by these agents is relatively short lived and therefore attenuates their cytolytic activity. The rise in Nrf-2 should therefore be considered as a chemoresistance mechanism to oxidative stress-inducing OXPHOS inhibitors. Combining these OXPHOS inhibitors with Nrf-2 modulators such as brusatol, results in a synergistic increase in inhibition of cancer cell proliferation [87]. Agents that enhance Nrf-2 activity are being developed to control oxidative damage in neurologic diseases. Similar efforts are needed to develop Nrf-2 inhibitors to enhance oxidative stress in HGSOC and other tumors.

Use of Nrf-2 inhibitors for cancer treatment also raises the possibility that such approaches may inhibit the natural protection against oxygen radicals in healthy tissues. Rational drug design, targeted delivery and specific drug formulations will be required to maximize the effect of Nrf-2 inhibitors in cancer cells while attenuating the side-effects of such drugs in healthy tissues.

### 5.3. Mitochondrial Adaptations to Oxidative Stress

The extensive use of atovaquone has led to the realization that some malarial parasites have developed resistance to this drug through mutations in cytochrome B (Cyt B), an essential component of complex III [97,98,99,100]. Cyt B is encoded by the mitochondrial genome. The mitochondrial population with mutated Cyt B is likely to be exposed to minimum oxidative damage in response to atovaquone and will therefore show enrichment through successive mitochondrial replications. A similar situation can also be envisioned in cancer where oxidative stress-inducing OXPHOS inhibitors may result in an increase in the drug-resistant mitochondrial pool. Studies are needed to determine the contributions of such mitochondrial adaptations to chemoresistance against OXPHOS inhibitors. 

## 6. Conclusions

Inhibition of glucose metabolism will result in significantly curtailing the ability of cancer cells to proliferate and modulate the tumor microenvironment through the release of lactic acid and other intermediates. OXPHOS pathway in tumors, cancer stem cells and the stromal and immune cells in the tumor microenvironment is recognized as a target for development of novel anti-cancer therapies. The multimeric complexes of the OXPHOS pathway are targets for small molecule inhibitors that can inhibit metabolism as well as induce oxidative damage and cancer cell death. OXPHOS inhibitors can be paired with immunologic and other therapies. While the development of novel OXPHOS inhibitors will be necessary, it will be important that these agents are specifically targeted to the cancer or the tumor microenvironment in order to reduce toxicity in healthy tissues. Finally, the use of OXPHOS inhibitors may result in Nrf-2 activation and mitochondrial adaptations that may pose as pathways that are typically not considered to contribute towards chemoresistance. Studies on atovaquone, proguanil, metformin have provided the foundation that will support the development of additional and more potent OXPHOS inhibitors for the treatment of HGSOC and other tumors.
